# Dental Biofilm and Saliva Microbiome and Its Interplay with Pediatric Allergies

**DOI:** 10.3390/microorganisms9061330

**Published:** 2021-06-18

**Authors:** Nicole B. Arweiler, Vivien Rahmel, Bilal Alashkar Alhamwe, Fahd Alhamdan, Michael Zemlin, Sébastien Boutin, Alexander Dalpke, Harald Renz

**Affiliations:** 1 Department of Periodontology and Peri-Implant Diseases, Philipps University Marburg, 35039 Marburg, Germany; rahmel@med.uni-marburg.de; 2 Institute of Tumor Immunology, Clinic for Hematology, Oncology and Immunology, Center for Tumor Biology and Immunology, Philipps University Marburg, 35039 Marburg, Germany; bilal.alashkaralhamwe@staff.uni-marburg.de; 3 Institute of Laboratory Medicine, Philipps University Marburg, Member of the German Center for Lung Research (DZL), 35039 Marburg, Germany; alhamdan@students.uni-marburg.de (F.A.); harald.renz@uk-gm.de (H.R.); 4 Department for General Pediatrics and Neonatology, Saarland University Medical Center, 66421 Homburg, Germany; michael.zemlin@uks.eu; 5 Department of Infectious Diseases, Medical Microbiology and Hygiene, University Heidelberg, 69120 Heidelberg, Germany; sebastien.boutin@med.uni-heidelberg.de; 6 Translational Lung Research Center Heidelberg (TLRC), German Center for Lung Research (DZL), University of Heidelberg, 69120 Heidelberg, Germany; 7 Institute of Medical Microbiology and Virology, University Hospital Carl Gustav Carus, Medical Faculty, Technische Universität Dresden, 01307 Dresden, Germany; alexander.dalpke@uniklinikum-dresden.de; 8 Department of Clinical Immunology and Allergology, Laboratory of Immunopathology, Sechenov University, Moscow, Russia, Member of Universities of Giessen and Marburg Lung Center (UGMLC), 35039 Marburg, Germany

**Keywords:** dental biofilm, saliva, oral microbiome, biofilm metabolism, 16s-rRNA gene next-generation sequencing

## Abstract

Little is known about the interplay and contribution of oral microorganisms to allergic diseases, especially in children. The aim of the clinical study was to associate saliva and dental biofilm microbiome with allergic disease, in particular with allergic asthma. In a single-center study, allergic/asthmatic children (*n* = 15; AA-Chd; age 10.7 ± 2.9), atopic/allergic children (*n* = 16; AT/AL-Chd; 11.3 ± 2.9), and healthy controls (*n* = 15; CON-Chd; age 9.9 ± 2.2) were recruited. After removing adhering biofilms from teeth and collecting saliva, microbiome was analyzed by using a 16s-rRNA gene-based next-generation sequencing in these two mediums. Microbiome structure differed significantly between saliva and dental biofilms (β-diversity). Within the groups, the dental biofilm microbiome of AA-Chd and AT/AL-Chd showed a similar microbial fingerprint characterized by only a small number of taxa that were enriched or depleted (4) compared to the CON-Chd, while both diseased groups showed a stronger microbial shift compared to CON-Chd, revealing 14 taxa in AA-Chd and 15 taxa in AT/AL-Chd that were different. This could be the first note to the contribution of dental biofilm and its metabolic activity to allergic health or disease.

## 1. Introduction

The oral microbiota constitutes an important part of the human microbiota and implies several hundred diverse species [1,2]. This microbiome is a normal part of the oral cavity and generally protects against colonization of extrinsic bacteria. While the oral microbiota in saliva is very similar to the planktonic phase, on non-shedding surfaces such as on teeth, restorations, oral implants, and between teeth, biofilms start to form. For many years, the accumulation of microorganisms on teeth and gingiva was called ‘dental plaque(s)’. Although it took some time to replace the name plaque with dental biofilm, it is evident that this organization of oral microorganisms fulfills all the criteria for a microbial biofilm [3] and can be subject to the so-called succession [4,5]. During seven days, a ‘primary flora’ dominated by streptococci changes to an anaerobic ‘climax community’, characterized by Gram-negative rods [6,7]. Due to different localizations and diverse exogenous influencing factors, plaque of different thickness and bacterial composition develops, not only on a macroscopic scale but also at the micro-ecological level, as related to O_2_-tension, local pH, matrix structure, and availability of nutritive substances [8]. In numerous studies, the spatial vitality state and three-dimensional structure, as well as the interplay of dental biofilms with underlying substrates, such as enamel, dentin, and dental restorations, have been extensively examined by using CLSM (confocal laser scanning microscopy) in combination with vitality fluorescence technique or FISH (fluorescence in situ hybridization) [7,9,10,11,12,13,14].

Dental biofilms and their sensitive ecosystem can turn out of balance and become a challenge for local (caries and periodontal diseases) and systemic health. An overgrowth by, e.g., periopathogenic species mostly in subgingival biofilm can provoke periodontal diseases like gingivitis and periodontitis, which are marked by chronic inflammation of the tooth-supporting structures with progressive alveolar bone loss [15]. As a consequence, clinical and inflammatory relationships between chronic periodontitis and other chronic metabolic, inflammatory, and vascular diseases, such as diabetes [16], cardiovascular diseases [17], chronic obstruction pulmonary disease (COPD) [18], metabolic syndrome, and obesity [19,20] were found, which is triggered not only by swallowed bacteria but rather by metabolic products of dental biofilms. In this context, the microbial community of dental biofilms has an important impact on the overall biology of humans.

However, only little is known about an association between dental biofilms and asthma or other allergic diseases in children. The few clinical findings—so far without a mechanistic insight and without microbiome analysis—are controversially discussed. Some studies showed a higher risk for caries lesions and higher prevalence in gingivitis in children with asthma compared to children without asthma [21,22,23,24], which are rather due to medication and not due the interplay with oral microflora. In contrast, Friedrich et al. [25] hypothesized that colonization by periodontal pathogens might (even) protect from allergic disease and found that respiratory allergies decreased with increasing periodontitis categories (however, in diabetes type 1 patients). Arbes and Matsui [26] raised the question of whether oral pathogens could positively influence allergic disease, and one study revealed that a certain pathogenetic oral microflora (in terms of dental diseases) transferred by parents through a pacifier could even be prevention against allergies [27]. Finally, a review revived the protective role of periodontal pathogens for asthma and other respiratory diseases (‘hygiene hypothesis’) but also stressed the need for further research [28]. These observations lead to the question of whether the oral microbiome and dental biofilm composition show an association with allergic diseases, in particular, with allergic asthma.

Thus, the study aimed to investigate differences in saliva and dental biofilm microbiome, including bacterial composition, diversity, and key taxa, between children suffering from allergic asthma or other allergies compared with healthy control children, and to shed light on the contribution of the dental biofilm microbiome signature to these diseases.

## 2. Materials and Methods

This observational, monocentric, clinically controlled trial was conducted in close interdisciplinary collaboration of dental, pediatric, and laboratory departments of the Philipps University and University Hospital Gießen and Marburg Ltd. in Marburg. The study protocol was reviewed and approved by the local Medical Ethics Committee (#24/07) in accordance with the latest revision of the declaration of Helsinki. 

### 2.1. Study Population

Children between the ages of 6 and 16 years were recruited to different study groups based on pulmologic and allergologic health status. After informed consent form was signed by subjects, parents, or legal guardians, data on personal information and current medication were collected. Allergic asthma was diagnosed by medical history, clinical examination, bodyplethysmography, positive skin prick test to aeroallergens, and/or specific serum IgE. Proven aeroallergens in AA-Chd were birch (5), hazelnut pollen (5), cat (4), dog (4), house dust mite (4), lychgrass (4), rye (4), beech (2), mugwort (2), oak (1). Atopic/allergic children (AT/AL-Chd) had sensitizations to wasp (4), rye (4), bee (3), hazelnut (3), house dust mite (2), birch (2), lychgrass (2), mugwort (1) and/or a positive family history of type 1 allergies. The control group of healthy children (CON-Chd) was recruited from the patient pool of the Dental Clinic and had no systemic diseases. None of the children included in the study received antibiotic treatment at least four weeks prior to sampling.

### 2.2. Dental Examination and Sampling Procedures

Study participants underwent dental examination in order to assess dental and gingival status. Dental parameters served as control parameters to confirm comparable dental and gingival conditions and were as follows: Saliva flow rate (SFR; ml/min), gingival bleeding index (GBI), and periodontal screening index (PSI), plaque control record by O´Leary (PCR) and dmft/DMFT, which assesses the number of decayed, missing, or filled teeth in first (lowercase letters) or second dentition (capital letters), or mixed. The presence of periodontitis or multiple carious lesions were criteria for exclusion. Care was taken that subjects restrained from tooth brushing and food intake at least two hours before sampling.

1. Saliva sampling, salivary flow rate, and other dental parameters

For the collection of unstimulated saliva, subjects were asked to spit 5 mL saliva directly in a 10 mL conical Falcon tube (Thermo Fisher Scientific Inc, Waltham, MA, USA). Salivary flow rate (SFR) was measured by measuring the time that it took to collect 5 mL and then calculated as mL/min. One milliliter of saliva was removed from the bottom of the vessel and placed into a 1.5 mL standard reaction tube (Eppendorf AG, Hamburg, Germany).

2. Dental biofilm sampling

Biofilm of all dental surfaces was harvested by using a soft toothbrush (Elmex Junior, CP GABA, Therwil, Switzerland) which was brushed 20 s on teeth. After that, tips of the bristles were cut off with a disposable surgical blade and transferred into a 1.5 mL reaction tube (Eppendorf AG, Hamburg, Germany). 

Dental biofilm and unstimulated saliva samples were subsequently stored at −80 °C.

### 2.3. Microbiome Analysis of Saliva and Dental Biofilm

- DNA isolation and sequencing

Bacterial DNA extraction from both substrates was performed by lysis, precipitation, and purification, which was conducted in a modified form according to protocols of the QIAamp^®^ DNA Mini Kit (QIAGEN N.V., Venlo, The Netherlands). The adapted method was tested and optimized by running several tests to ensure its suitability. DNA yield and extraction process quality were determined afterwards by the measurement of absorbance to monitor if concentration of genetic material and quality of samples were sufficient for DNA sequencing by using NanoDrop™ 2000 full-spectrum UV/VIS spectrophotometer (Thermo Fisher Scientific Inc., Waltham, MA, USA).

- 16s rRNA gene data collection and sequencing

DNA was amplified using universal bacterial primers 515F and 806R targeting the V4 region of the 16S rRNA gene [29]. The primers were modified to include a unique barcode and Illumina primers sequences (P5 and P7). PCR reactions were performed as previously described, including negative control and positive controls (mock community, HM-782D, Bei resources) [30]. Library was prepared by PCR with 5 cycles using homemade primers combining the Illumina sequencing primers and the Illumina sequencing adapters and then purified by using Agencourt AMPure XP beads (Beckman Coulter, 47807 Krefeld, Germany) following the manufacturer’s instructions. Purified products were checked for quality and concentration using Quant-iT™ PicoGreen^®^ dsDNA Assay Kit (ThermoFisher Scientific GmbH, Dreieich, Germany) and Qiaxcel instrument (QIAGEN GmbH, Hilden, Germany). An equimolar mix of all the PCR products was then sequenced on an Illumina Miseq instrument (V3 chemistry, 2 × 300 bp).

- Microbiome sequencing analysis

Raw reads were processed using dada2 (version 1.16.0) to denoise quality filter reads (maximum ambiguity: 0, number of expected errors for each read: 1, truncate reads at the first instance of a quality score less than 2) and call amplicon sequence variants (ASVs), and a feature table of ASV counts was generated. After quality filtering, reads were merged as contigs and checked for chimera with the default parameters. Sequence data were compared against a SILVA reference database (Version 132) and bacterial taxonomies were assigned to the ASV feature table. Beta-diversity among samples was explored via multi-dimensional approaches (Principal coordinate’s analysis) and PERMANOVA. DESeq2 (version 1.29.6) was used to identify taxa displaying differences in abundance between groups. All data analyses were performed using R software v 4.0.

## 3. Results

### 3.1. Demographic Information and Dental Parameter 

Table 1 shows the basic characteristics of the study population with no significant differences in age, sex, and BMI (*p* > 0.05). Dental parameters SFR, dmft/DMFT index periodontal screening index (PSI), gingival bleeding index (GBI), and plaque control record (PCR) are also depicted in Table 1. All indices served as control parameters to confirm similar dental and gingival conditions. They were significantly different in GBI (CON-Chd compared to AA-Chd and AT/AL-Chd; *p* < 0.01, as well as between AA-Chd and AT/AL-Chd; *p* < 0.05) and in PSI (AT/AL-Chd compared to CON-Chd, *p* < 0.01), which was, however, clinically not relevant. PSI was in all groups around 1 corresponding to gingival bleeding, no child had signs of periodontitis. 

### 3.2. Comparison of Saliva and Biofilm 

**Figure 1:**
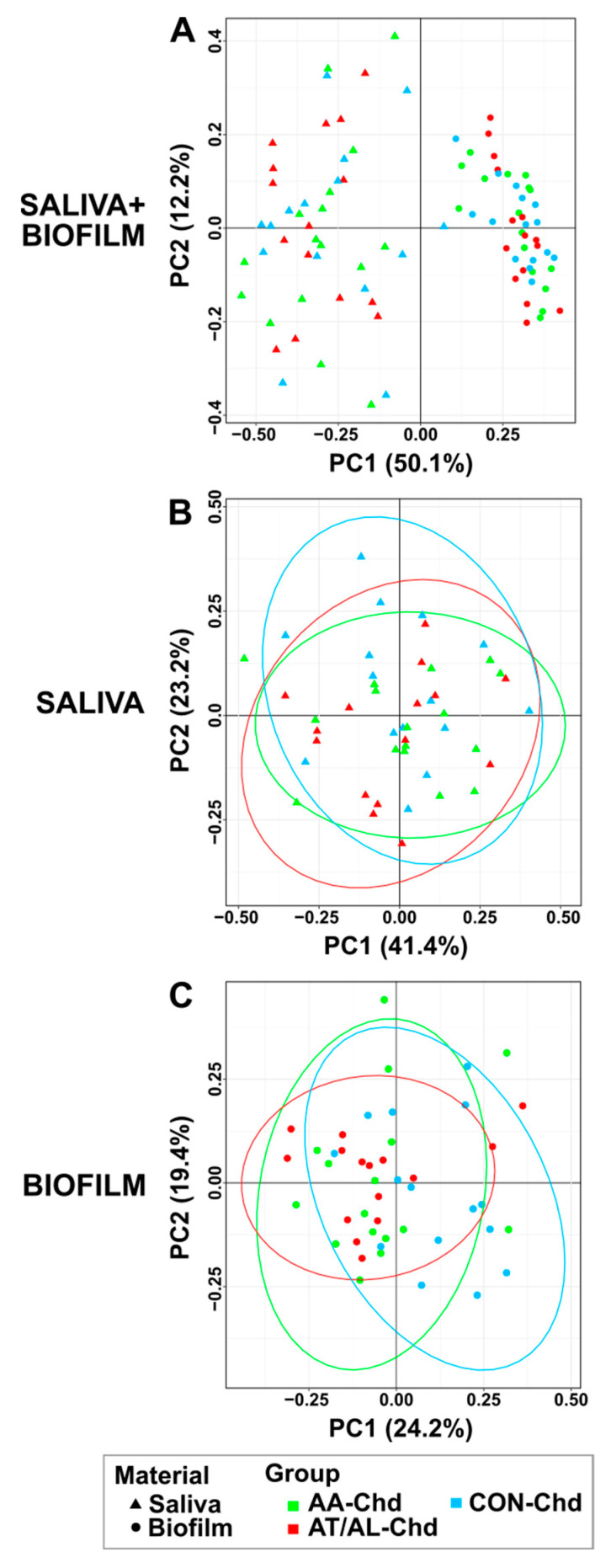
PCoA plot showing the relatedness between the microbiota’s structure of AA-Chd (green), AT/AL-Chd (red) and CON-Chd (blue) for both saliva and biofilm (**A**), saliva alone (**B**), and biofilm alone (**C**). The PCoA plots were based on the Morisita–Horn distance. Ellipses represent the 95% confidence ellipse based on a multivariate t-distribution.

The β-diversity of the microbiome analysis is visualized in Figure 1. Significant differences in the microbiome structure were found comparing the two habitats: Saliva and biofilm (Figure 1A; using PERMANOVA model; r^2^ = 0.17, *p* < 0.001). Saliva β-diversity revealed no significant differences when comparing pediatric groups (Figure 1B), while dental biofilm β-diversity revealed highly similar microbiome structure between the AA-Chd and AT/AL-Chd (r^2^ = 0.02, *p* = 0.796), which was distinct from the CON-Chd (AA-Chd: r^2^ = 0.09, *p* = 0.018; AT/AL-Chd: r^2^ = 0.08, *p* = 0.021) (Figure 1C). 

**Figure 2:**
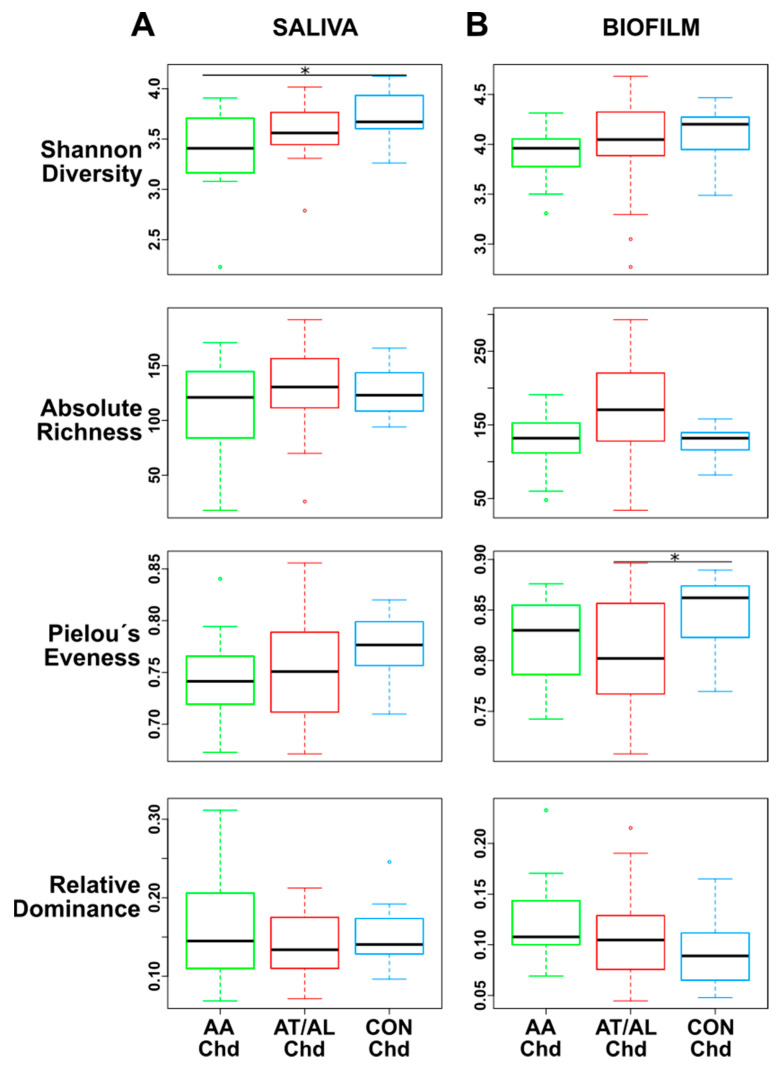
α-diversity metrics: Shannon diversity index, absolute richness, Pielou´s eveness index, and relative dominance of saliva (**A**) and biofilm (**B**) samples in the different groups presented as boxplots (* *p* < 0.05; by Wilcoxon rank sum tests).

In terms of α-diversity (Figure 2), the saliva microbiome revealed a significant increase of diversity (Shannon index) in the CON-Chd compared to AA-Chd, but no significant differences with regard to richness, Pielou´s evenness, and dominance (Figure 2A). 

When comparing groups in dental biofilm, α-diversity indicated a slightly higher Shannon index in the CON-Chd group compared to diseased groups (similar to saliva) but without statistical significance. Only Pielou’s evenness in CON-Chd compared to AT/AL-Chd (Figure 2B) reached the level of statistical significance.

### 3.3. Enriched or Depleted Taxa of Dental Biofilm 

**Figure 3:**
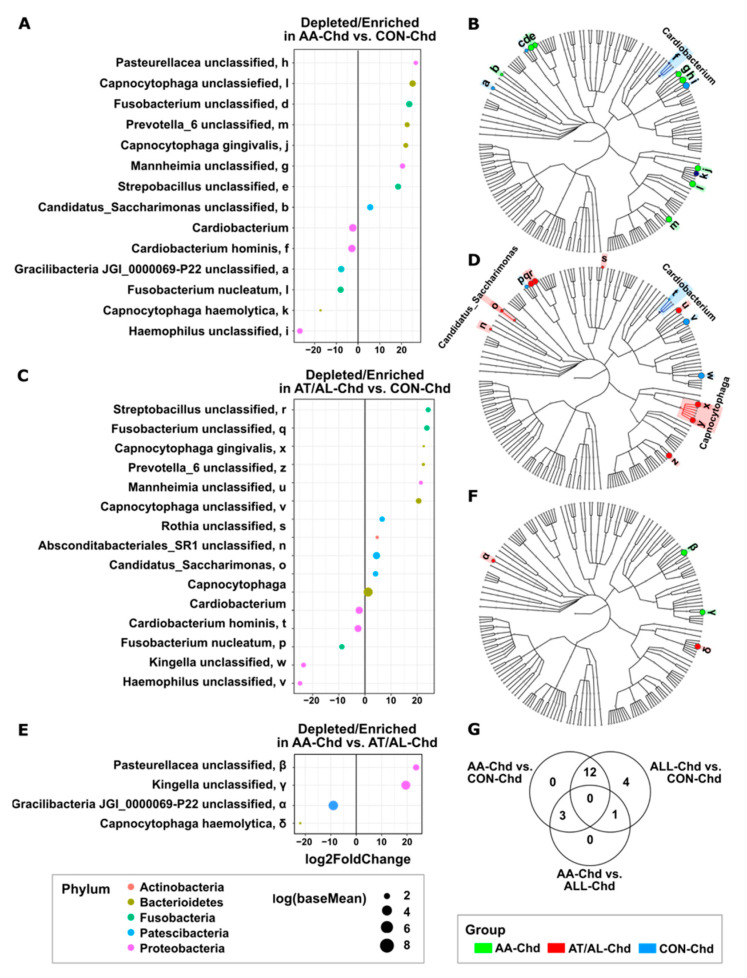
Differentially abundant taxa between the groups in biofilms. (**A**) Log2 fold change between AA-Chd and CON-Chd, (**B**) between AT/AL-Chd and CON-Chd (**C**), as well as between AA-Chd and AT/AL-Chd (**E**). Cladograms showing the most differentially abundant taxa enriched in microbiota from AA-Chd (green) and CON-Chd (blue) (**B**), AT/AL-Chd (red) and CON-Chd (blue) (**D**) as well as AA-Chd (green) and AT/AL-Chd (red) (**F**). (**G**) Venn diagram illustrating unique and shared species among child group comparisons.

It could be shown that certain taxa were differentially enriched or depleted between AA-Chd and CON-Chd (Figure 3A,B), AT/AL-Chd and CON-Chd (Figure 3C,D), and AA-Chd and AT/AL-Chd (Figure 3E,F). The comparison of AA-Chd versus CON-Chd revealed fourteen taxa that were enriched or depleted, while fifteen taxa were different in comparison of AT/AL-Chd and CON-Chd (Figure 3G). A major overlap could be observed between the differentially abundant taxa of AA-Chd and AT/AL-Chd compared to CON-Chd. In both diseased groups, the same ribosomal sequence variants belonging to Capnocytophaga gingivalis and the genus Capnocytophaga, Fusobacterium, Prevotella_6 and Mannheimia were enriched, and Cardiobacterium hominis, Fusobacterium nucleatum and Haemophilus sp. were depleted. Furthermore, the biofilm microbiome between AA-Chd and AT/AL-Chd differed by only four taxa: Pasteurellaceae unclassified and Kingella unclassified were enriched in AA-Chd, and Capnocytophaga haemolytica and Gracilibacteria P22 unclassified were depleted, respectively (Figure 3E,F). 

## 4. Discussion

The present study aimed to find a typical fingerprint in oral microbiome of two pediatric groups with allergic phenotypes, namely allergic asthmatic and atopic/allergic children, in comparison with healthy children, thus not suffering from asthmatic, atopic, or allergic diseases.

Notably, multiple factors, including antibiotics, birth method, breastfeeding, dental caries, and periodontitis, may impact the composition and diversity of the microbiome, which is potentially contributing to the development of allergic disease in childhood, such as asthma [31,32,33,34,35]. There is accumulating evidence reflecting a strong correlation between early-life microbial dysbiosis and allergy development during childhood [36,37]. Most of the studies present today are focusing on the correlation between gut, skin, and respiratory tract microbiome dysbiosis and the development of allergy, such as asthma [38]. However, the oral cavity is the initial interface between allergens, microbiome, and mucosal immunity and, in this aspect, relatively little describes the interactions between the oral microbiome dysbiosis and allergy development. 

The microbiome profiles revealed distinct microbial and metabolic signatures correlated to mucosal immune disturbances in allergic diseases, such as asthma or food allergy, in children and adults, respectively [39,40]. In addition, some oral pathogens exert the ability to influence the plasticity of immune cells, such as macrophages in periodontal tissue [41]. Highlighting the role of oral microbiome dysbiosis in saliva and dental biofilm as an anatomic site of interest should significantly enhance our deciphering of its influence on allergic diseases.

Analysis of α- and β-diversity of the saliva microbiome found no significant differences between study groups, and thus, it was focused only on biofilm concerning further taxa analysis. This is in line with former data considering that saliva represents a planktonic phase of the oral microbiota and does not have its own resident microbiota [8]. Saliva contains, similar to bacterial laboratory fluid cultures, a high number of bacteria (up to 10^9^ microorganisms per milliliter) but is swallowed continuously and about 5 g of bacteria ‘disappear’ into the stomach daily. In contrast to dental plaque biofilm, bacterial numbers in saliva do not multiply within the mouth [8], but it should be kept in mind, that saliva is the primary source for the continuous bacterial (re)colonization of the diverse oral soft and hard surfaces and biofilms are formed by bacteria supplied by saliva microbiome. Dental biofilm undergoes different phases, which are characteristic during succession and also holds true for all other natural occurring biofilms—such as medical or environmental biofilms. Shortly after the first induction phase characterized by the formation of the pellicle (‘conditioning film’ or ‘linking film’) that comes from saliva [42], accumulation of pioneer bacteria occurs followed by a third (‘existence’) phase. These mature biofilms that adhere to assorted natural or artificial surfaces usually exist in aqueous environments containing a sufficient concentration of nutrients to sustain the metabolic needs of the microbiota. Therefore, they harbor a plethora of bacterial species, and thus, are extremely heterogeneous [43,44]. This complex and highly organized community, that is also named ‘city of microbes’ [45], does not only interact with its underlying substrate but also with its host at different sites through different pathways by swallowing, systemic inflammation, and systemic bacterial dissemination [46]. Many studies have dealt with spatial distribution, altered growth rate, and antibiotic resistance of oral biofilms [9,10,11,12,13,14,47]. However, concomitant with modern possibilities to analyze the microbiome (in particular by using next-generation sequencing), it is of high interest to also explore the influence of this biofilm affecting systemic health [15].

It was a striking finding that the microbial composition between allergic asthmatic children and atopic/allergic children differed only in four taxa, while both diseased groups revealed many more depleted/enriched taxa as compared to the healthy counter group. It is already established that during childhood, bacterial diversity of oral cavity arises with eruption of teeth since hard surfaces enable the development of dental biofilms [15]. Throughout childhood, the oral microbiome is a highly dynamic community undergoing multiple changes in composition towards a more stable ecosystem in adolescents [5]. While early-life exposure to environmental microbiota seems to play an important role in providing anti-inflammatory, anti-allergic, and pro-tolerogenic signals to the host immune system [48], in this cross-sectional study, the contribution of the special taxa that were depleted or enriched is, at this stage, completely unclear.

Although key bacteria, *Porphyromonas gingivalis* and *Aggregatibacter actinomycetemcomitans,* that were formerly marked in relation to hygiene hypothesis and periodontitis [26,28,39], were not accounted for differences, the results still indicate that obviously allergy induces a strong shift in the biofilm’s microbial composition. In terms of periopathogens, healthy children (CON-Chd) had enrichment of *Fusobacterium unclassified* and *Prevotella_6* unclassified, while *Fusobacterium nucleatum* was depleted. Due to the uniqueness of this finding in pediatric biofilms and the lack of similar studies, these results are difficult to classify and discuss. Larger sample size and a cause-related and mechanistic insight into the contribution of dental biofilm to disease development are now required. This could allow subanalysis, how differences in system metabolism (or medication) between these healthy and diseased groups could contribute to or prevent allergic/atopic diseases. 

Nevertheless, this cross-sectional analysis with a relatively small number of subjects could give the first and valuable hint for further analysis. Furthermore, it needs to be examined whether this microbial fingerprint precedes disease development and can possibly be influenced. 

## 5. Conclusions

The oral microbiome is a highly dynamic community affected by multiple environmental factors with a complex interplay of cellular interactions in allergic diseases, such as asthma. The present data demonstrated a shift in dental biofilm’s microbial composition in allergic groups, indicating a possible contribution of dental biofilm to allergy and asthma in children. Interestingly, periopathogenetic bacteria *Fusobacterium nucleatum* was enriched in allergic groups, while Fusobacterium unclassified and Prevotella_6 unclassified were depleted compared to healthy children (CON-Chd). 

The analysis indicates that dental biofilm microbiome is informative for future studies, especially in discovering biologically plausible mechanisms by which oral pathogens (including periopathogens) could influence the risk of allergic disease. 

## Figures and Tables

**Table 1:** Demographic information of study participants (mean ± standard deviation, SD) and dental parameters.

Characteristic	AA-Chd	AT/AL-Chd	CON-Chd
Group size	*n* = 15	*n* = 16	*n* = 15
Age (years)	10.73 ± 2.89	11.31 ± 2.82	9.93 ± 2.22
Sex (male:female)	8:7	8:8	9:6
BMI (kg/m^2^ mean)	18.79 ± 2.44	19.49 ± 5.63	17.66 ± 4.07
SFR (mL/min)	0.33 ± 0.18	0.53 ± 0.25	0.28 ± 0.16
Dmft/DMFT	0.47 ± 1.30	1.00 ± 2.00	1.27 ± 1.91
PSI	0.73 ± 0.45	0.41 ± 0.39 ^a^	1.07 ± 0.38 ^a^
GBI (%)	16.00 ± 13.00 ^a,b^	8.00 ± 9.00 ^a,b^	36.00 ± 12.00 ^a^
PCR (%)	68.00 ± 27.00	65.00 ± 27.00	51.00 ± 11.00

^a^: Significantly different (*p* < 0.05) compared to CON-Chd, ^b^: Significant difference between AA-Chd and AT/AL-Chd by Wilcoxon rank sum test.

## Data Availability

10.6084/m9.figshare.14578518.
